# Cholesterol-dependent LXR transcription factor activity represses pronociceptive effects of estrogen in sensory neurons and pain induced by myelin basic protein fragments

**DOI:** 10.1016/j.bbih.2024.100757

**Published:** 2024-03-30

**Authors:** Swathi K. Hullugundi, Jennifer Dolkas, Andrei V. Chernov, Tony L. Yaksh, Kelly A. Eddinger, Mila Angert, Glaucilene Ferreira Catroli, Alex Y. Strongin, Patrick M. Dougherty, Yan Li, Oswal Quehenberger, Aaron Armando, Veronica I. Shubayev

**Affiliations:** a Department of Anesthesiology, University of California, San Diego, La Jolla, CA, USA; b VA San Diego Healthcare System, La Jolla, CA, USA; c Sanford Burnham Prebys Medical Discovery Institute, La Jolla, CA, USA; d Department of Pain Medicine, University of Texas MD Anderson Cancer Center, Houston, TX, USA; e Lipidomics Core, University of California, San Diego, La Jolla, CA, USA

**Keywords:** Neuropathic pain, Oxysterol, Cholesterol, Liver x receptor, LXR, Myelin basic protein, Estrogen, Interleukin 6, DRG culture, Sensory neuron

## Abstract

**Background:**

A bioactive myelin basic protein (MBP) fragment, comprising MBP_84-104_, is released in sciatic nerve after chronic constriction injury (CCI). Intraneural injection (IN) of MBP_84-104_ in an intact sciatic nerve is sufficient to induce persistent neuropathic pain-like behavior via robust transcriptional remodeling at the injection site and ipsilateral dorsal root ganglia (DRG) and spinal cord. The sex (female)-specific pronociceptive activity of MBP_84-104_ associates with sex-specific changes in cholesterol metabolism and activation of estrogen receptor (ESR)1 signaling.

**Methods:**

In male and female normal and post-CCI rat sciatic nerves, we assessed: (i) cholesterol precursor and metabolite levels by lipidomics; (ii) MBP_84-104_ interactors by mass spectrometry of MBP_84-104_ pull-down; and (iii) liver X receptor (LXR)α protein expression by immunoblotting. To test the effect of LXRα stimulation on IN MBP_84-104_-induced mechanical hypersensitivity, the LXRα expression was confirmed along the segmental neuraxis, in DRG and spinal cord, followed by von Frey testing of the effect of intrathecally administered synthetic LXR agonist, GW3965. In cultured male and female rat DRGs exposed to MBP_84-104_ and/or estrogen treatments, transcriptional effect of LXR stimulation by GW3965 was assessed on downstream cholesterol transporter Abc, interleukin (IL)-6, and pronociceptive Cacna2d1 gene expression.

**Results:**

CCI regulated LXRα ligand and receptor levels in nerves of both sexes, with cholesterol precursors, desmosterol and 7-DHC, and oxysterol elevated in females relative to males. MBP_84-104_ interacted with nuclear receptor coactivator (Ncoa)1, known to activate LXRα, injury-specific in nerves of both sexes. LXR stimulation suppressed ESR1-induced IL-6 and Cacna2d1 expression in cultured DRGs of both sexes and attenuated MBP_84-104_-induced pain in females.

**Conclusion:**

The injury-released bioactive MBP fragments induce pronociceptive changes by selective inactivation of nuclear transcription factors, including LXRα. By Ncoa1 sequestration, bioactive MBP fragments render LXRα function to counteract pronociceptive activity of estrogen/ESR1 in sensory neurons. This effect of MBP fragments is prevalent in females due to high circulating estrogen levels in females relative to males. Restoring LXR activity presents a promising therapeutic strategy in management of neuropathic pain induced by bioactive MBP.

## Highlights

• Cholesterol precursor and derivative ligands of LXR are regulated in nerve post-injury.
• MBP fragments released in nerve post-injury bind a nuclear receptor co-activator Ncoa1.
• LXR activation controls pro-nociceptive activity of estrogens in sensory neurons and pain induced by MBP fragments.

## 1 Introduction

Lipid-rich myelin sheath provides insulation to primary mechanosensory (A-type) dorsal root ganglia (DRG) neurons in the peripheral nervous system (PNS). An injury-induced loss of the structural and molecular integrity of PNS myelin may lead to neuropathic pain secondary to a normally innocuous tactile stimulus (mechanical allodynia). Multilamellar organization of myelin sheath depends in part on electrostatic interactions between its cationic myelin basic protein (MBP) and anionic lipids (Boggs, 2006; Harauz and Boggs, 2013). Loss of myelin integrity results in aberrant insertion of ion channels and ectopic excitability of A-afferents (Devor, 2009; Wu et al., 2002; Zhu et al., 2012). In addition, traumatic PNS injury such as sciatic nerve chronic constriction injury (CCI) causes proteolytic release of bioactive pronociceptive MBP fragments such as MBP_84-104_ (Chernov et al., 2018; Hong et al., 2017; Ko et al., 2016; Kobayashi et al., 2008; Lee et al., 2022; Liu et al., 2012; Remacle et al., 2018), as illustrated (Fig. 1A).

**Fig. 1:**
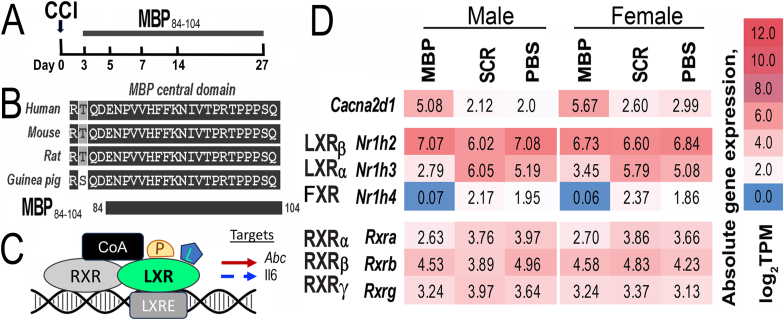
***MBP***_***84-104***_***suppresses LXRα/RXRα axis in sciatic nerve.A****, The degraded (d)MBP fragment comprising the MBP*_*84-104*_*epitope is released in nerve between days 3 and 27 post-CCI (**Lee et al., 2022**;**Liu et al., 2012**) (a schematic).****B,****Sequence alignment of the MBP central domain showing strong sequence conservation with 100% identity between human and rodent MBP*_*84-104*_*regions.****C,****The LXR/RXR axis (a schematic). Upon activation by cholesterol precursor (P) or oxysterol ligand (L), LXR dimerizes with RXR, binds to LXR-responsive-element (LXRE) on a target gene promoter leading to induction (red arrow) of Abc cholesterol transporter or suppression (blue arrow) of IL-6 expression.****D****, Heat maps of normalized absolute gene expression values (Log2TPMs, transcripts per million reads) for RNA-seq dataset (GEO ID**GSE107159**) obtained in sciatic nerve (injection site) after IN MBP*_*84-104*_*or SCR peptide or PBS in mice in n=12/group (6 male, 6 female (**Chernov et al., 2020**). Gene subsets were selected based on Gene Ontology annotations for LXR/Nr1h, RXR/Rxr and Cacna2d1 genes*. (For interpretation of the references to color in this figure legend, the reader is referred to the Web version of this article.)

MBP_84-104_ is a highly evolutionarily conserved motif (identical in humans and rodents, Fig. 1B) comprising an immunodominant T cell epitope implicated in the pathogenesis of autoimmune demyelinating disease, multiple sclerosis (Harauz and Boggs, 2013). Intraneural injection of MBP_84-104_ peptide into an intact sciatic nerve (IN MBP_84-104_) is sufficient to initiate a state of mechanical allodynia sustained for up to 4 weeks (Chernov et al., 2018; Hong et al., 2017; Ko et al., 2016; Liu et al., 2012; Remacle et al., 2018). Pronociceptive activity of IN MBP_84-104_ is T cell-dependent, as shown using athymic nude rats (Liu et al., 2012), charge-dependent, as shown by mutagenesis of the peptide's H89 site (Chernov et al., 2018), and associates with transcriptional remodeling of lipid energy metabolism (Chernov et al., 2020).

In the PNS, lipid energy expenditure is regulated via a hierarchical nuclear transcription factor network that includes liver X receptors (LXRs) activated by cholesterol derivatives such as oxysterols and precursors such as desmosterol (Bookout et al., 2006). The LXR family consists of two isotypes, LXRα (encoded by *Nr1h3*) and LXRβ (encoded by *Nr1h2*) (Repa and Mangelsdorf, 2000). LXRs form obligate heterodimers with the retinoid X receptor (RXR)α, and recruit co-activators such as nuclear receptor co-activator (Ncoa1, also known as steroid receptor coactivator (Src)1), resulting in the induced transcription of the target reverse cholesterol transporter/efflux genes such as ATP binding cassette (Abc) transporters A1 (Abca1) and G1 (Abcg1), and repression of inflammatory genes such as interleukin (Il)*6* (Castrillo et al., 2003; Joseph et al., 2003; Li et al., 2019; Storti et al., 2019; Willy et al., 1995), as illustrated (Fig. 1C).

Loss-of-function experiments have implicated LXRα, not LXRβ, in preventing the development of mechanical allodynia caused by PNS injury due to trauma (Xu et al., 2017), diabetic neuropathy (Cermenati et al., 2010), and obesity (Gavini et al., 2018). LXR agonist treatment mitigated mechanical allodynia through the restoration of cholesterol efflux and the reversal of the endoplasmic reticulum (ER)-stress in DRG neurons and/or spinal cord (Bao et al., 2017; Cermenati et al., 2010; Gavini et al., 2018; Li et al., 2019; Xu et al., 2017). IN MBP_84-104_ sustains mechanical allodynia through activation of ER-stress and IL-6 activity in DRG neurons and/or spinal cord (Chernov et al., 2020; Ko et al., 2016). IN MBP_84-104_ also selectively suppresses LXRα and RXRα, but not LXRβ and other RXR genes (Chernov et al., 2020) and (Fig. 1D).

Females are more prevalent sufferers of chronic pain relative to males (Fillingim et al., 2009; Mogil, 2012; Nahin, 2015; Sorge et al., 2015; Yu et al., 2020). IN MBP_84-104_ causes mechanical allodynia exclusively in female, not in male, mice (Chernov et al., 2020). This IN MBP_84-104_ action has been linked to activation of ESR1 signaling in female relative to male DRG and/or spinal cord. While complex actions of estrogens in pain are still debated (Amandusson et al., 1995; Khomula et al., 2017; Papka et al., 1997, 2001; Tran et al., 2017, 2020; Vanderhorst et al., 2005), ESR1 contributes to persistent pain by augmenting Ca^2+^-dependent ER-stress in DRG neurons (Ferrari et al., 2016). LXR stimulation partially inactivates ESR1 in other systems (DuSell et al., 2008). The present study established the relationship between the cholesterol- and estrogen-dependent nuclear receptor systems in a context of somatosensory system, PNS injury and bioactive activity of pronociceptive MBP fragments.

## 2 Materials and methods

### 2.1 Reagents and antibodies

Common reagents, the LXRα/β agonist, GW3965 (G6295), and 17β-estradiol (E2758) were obtained from MilliporeSigma. ESR1 agonist 4,4′,4'-(4-Propyl-[1H]-pyrazole-1,3,5-triyl) trisphenol (PPT) was obtained from Tocris (1426). MBP_84-104_ (ENPVVHFFKNIVTPRTPPPSQ) and scrambled (SCR; NKPQTNVVEPFHRTFPIPVS) peptides, protected by N-terminal acetylation and C-terminal amidation, were synthesized by GenScript based on the human MBP sequence (AAH08749, GenBank). The following *primary antibodies* used for immunodetection: mouse anti-CD68 (MLA341R, Serotec, 1:100); rabbit anti-ESR1 (MA1-27107, Invitrogen, 1:50); mouse anti-flotillin-1 (610820, BD Biosciences, 1:700); mouse anti-GAPDH (glyceraldehyde 3-phosphate dehydrogenase, 32233, Santa Cruz Biotechnology, 1:2000); rabbit anti-LXRα (ab176323, Abcam, 1:100); rabbit anti-degraded MBP (dMBP, AB5864, 1:2000); mouse anti-Ncoa1/Src1 (LS-B1702, LSBio, 1:50); rabbit anti-ESR1 (06–935, 1:400); mouse anti-GFAP (glial fibrillary acidic protein, MAB360, 1:400); mouse anti-NF200 (neurofilament 200, N0142, 1:400); and mouse anti-NeuN (MAB377, 1:1000) are all from MilliporeSigma.

### 2.2 Animals and tissues

Adult female and male Sprague-Dawley rats (Envigo, 8–10-week-old) were housed in plastic cages in temperature- and light-controlled (12-h light/dark cycle) conditions with ad libitum access to food and water. All procedures were conducted during the light cycle. Animals were randomly assigned to the surgical and treatment groups. All animal procedures were performed in accordance with the National Institutes of Health Guide for the Care and Use of Laboratory Animals, the ethical guidelines of the International Association for the Study of Pain, and the experimental protocols approved by the Institutional Animal Care and Use Committee (IACUC) at the University of California, San Diego, the Veterans Affairs San Diego Healthcare System, and the University of Texas MD Anderson Cancer Center. All procedures were designed to minimize animal discomfort and studies were powered to use the fewest possible number of animals.

### 2.3 Animal models

Under isoflurane anesthesia (4% induction, 2% maintenance, Isothesia, Henry Schein), the common sciatic nerve was exposed unilaterally (left side) at the mid-thigh level through a gluteal muscle-splitting incision. Using sterilized micro scissors and Jewelers forceps from Roboz, *chronic constriction injury (CCI)* was administered by applying three loosely constrictive 5.0 chromic gut ligatures (Ethicon suture 634G) around the sciatic nerve (Bennett and Xie, 1988). Care was taken to produce consistent ligations that constricted no more than one-third of the diameter of the nerve, using a double knot. In a separate group of female rats, a single bolus *intraneural (IN) injection of the MBP*_*84-104*_ (IN MBP_84-104_) or SCR peptides (50 μg in 5 μl phosphate buffered saline (PBS), each) or PBS alone (5 μl) was injected into the sciatic nerve fascicle, using a 33-gauge needle on a Hamilton syringe, as previously described (Chernov et al., 2018; Ko et al., 2016; Liu et al., 2012). In sham operated rats, the left sciatic nerve was exposed, but not ligated. In all groups, muscle was sutured closed using 4.0 perma-hand silk suture (Ethicon 683G) and skin was stapled closed. Aside from isoflurane, no additional analgesics were given.

### 2.4 Tissues collection

For immunoblotting, lipidomics and mass spectrometry analyses, rats were sacrificed by intraperitoneal Euthasol (100–150 mg/ml; Virbac Animal Health). In CCI rats, Ethicon sutures were removed prior to sciatic nerve excision. Tissues were excised, snap-frozen in liquid nitrogen and stored at −80 °C. For immunostaining, rats were perfused transcardially under deep anesthesia with a saline flush followed by fresh 4% paraformaldehyde (PFA) in 0.2 M phosphate buffer. Sciatic nerve injection, CCI injury (3 mm distal and 3 mm proximal to the ligature location) or anatomically equvalent sites, lumbar (L)4–5 dorsal root ganglia (DRG) and spinal cord lumbar enlargement (L1-L6) tissues were processed for analyses. Tissues contralateral to injury or IN injection, or from sham-operated or naïve animals, were collected for controls.

### 2.5 IT LXR agonist therapy

Intrathecal (IT) catheters were placed and secured, as reported (Malkmus and Yaksh, 2004). In brief, under isoflurane inhalant anesthesia, each animal was given subcutaneous Lactated Ringer's Solution (20 mL/kg) with added carprofen (5 mg/kg). The back of the head and neck was then shaved, and the animal mounted into a stereotaxic unit. The surgical site was prepped with alternating applications of commercially available chlorhexidine gluconate solution and 70% isopropyl alcohol. Using a 15# scalpel blade, a 1 cm skin incision was made along the dorsal midline of the skull, being careful not to incise any of the underlying muscle. With the scalpel, starting from the nuchal crest, the levator auris muscle was gently dissected away from the occipital crest. The muscle was further separated from the crest using a periodontal elevator, taking care not to shred or split the muscle body. The muscle and skin were retracted to expose the cisternal membrane. A 22G needle with the bevel bent to 45° was used to make a small incision (1 mm) through the dura, whereon cerebral spinal fluid began to pulse from the incision. The prepared catheter was steadily fed down the intrathecal space to a length of 8.5 cm, watching for any muscle twitching or spasms. If twitching or spasms were observed, the catheter was retracted, redirected, and refed until it could be fed without issue. Upon placement, the catheter was flushed with 10 μL NaCl 0.9% and again observed for any signs of twitching or spasms. If twitching, spasms, or retraction of an extremity were observed during the flush, the catheter was removed and re-fed until free of deleterious effects. The catheter was externalized through the skin of the skull, between the ears. A metal plug was fitted, and the incision closed in a single layer, using one of the skin sutures to secure the muscle in place. During that same anesthetic session, each animal was given IN MBP_84-104_ (above). At day 7 after intrathecal catheter implant and IN, animals received a single IT injection of LXR agonist, GW3695 (6.2 μg/10 μl), or vehicle (1% DMSO and 4% Tween-80 in PBS), as published (Xu et al., 2017).

### 2.6 von Frey testing

Sensitivity to non-noxious mechanical stimuli was measured using a von Frey test. Rats were habituated to the testing environment prior to baseline tests and randomly assigned to experimental groups. Testing was performed during the light cycle by an experimenter blinded to the treatment groups. Rats were placed in individual Plexiglas compartments with a wire mesh bottom. The plantar surface of the hind paw within the sciatic nerve innervation area was stimulated using calibrated von Frey filaments (Stoelting, Wood Dale, IL, USA). Stimuli were applied for 4–6 s with a 0.4–15.0 g buckling force to the mid paw plantar surface with ascending filament stiffness until a paw withdrawal response occurred. Stimuli were separated by several-second intervals or until the animal was calm with both hind paws placed on the grid. The consecutive way of applying filaments was continued until six responses were recorded. The 50% threshold was calculated using Dixon's up-down method (Chaplan et al., 1994).

### 2.7 Acute dissociation of DRG neurons

Thoracic and/or lumbar ganglia were dissected bilaterally from deeply anesthetized rats (Envigo, 6–8-week-old, female and male), enzymatically dissociated in low-glucose DMEM containing 1% collagenase and 2.5% trypsin and plated onto poly-D-lysine (PDL) and laminin (Sigma) coated plates in neurobasal medium (Gibco) supplemented with B27 Gem21 Neuroplex (Gemini Bioproducts), NGF (25 ng/ml; Sigma) and glutamine (2 mM; Gibco) at 37 °C, 5% CO_2_, as reported (Remacle et al., 2018). Cells were plated in the neurobasal medium for treatment (below) or fixed in 4% PFA for immunostaining. *For calcium imaging*, bilateral L4-L5 ganglia were plated in a Petri dish containing trypsin (0.0625 mg/ml, Hyclone) and type IA collagenase (1 mg/ml, Sigma-Aldrich) in DMEM and shaken for 50 min in a heated (37 °C) bath. The cells were washed, mechanically dispersed with a fire polished Pasteur pipette, plated on PDL-coated glass sheets and held in culture dishes with DMEM (10% FBS) (Li et al., 2018).

### 2.8 DRG culture treatment

GW3965, 17β-estradiol and PPT were dissolved in DMSO and frozen in aliquots. Reagents were diluted in PBS prior to adding them to the cultures. Dissociated DRG cells were plated, followed by: (a) pre-treatment with GW3965 (1 μM) and/or 17β-estradiol (100 nM) for 24 h, followed by the incubation with MBP_84-104_ or SCR peptide (10 μg/ml, each) for 24 h; or (b) incubation with MBP_84-104_ (10 μg/ml), 17β-estradiol (100 nM) or PPT (100 nM) for 16–18 h, followed by incubation with GW3965 (1 μM) and fresh MBP_84-104_ (10 μg/ml), 17β-estradiol or PPT (100 nM, each) for 24 h. Lysates from 2 to 3 independent experiments from 10 to 15 animals per sex, each, corresponding to 12–15 plates per experiment, were collected in Trizol for qPCR analysis.

### 2.9 Intracellular calcium imaging

Dissociated DRG cells were loaded with the ratio metric Ca^2+^ indicator dye Fura-2-acetoxymethyl ester (2 μM; Molecular Probes) for at least 40 min at 37 °C in 10 mM HEPES (pH 7.4) containing 140 mM NaCl, 5 mM KCl, 2 mM CaCl_2_, 2 mM MgCl_2_, and 11 mM glucose (extracellular solution). The cells were then transferred to a recording chamber placed on a microscope (Nikon Eclipse) and continuously perfused with the oxygenated (95% O_2_ and 5% CO_2_) extracellular solution (2 ml/min) at ambient temperature. The intracellular calcium concentration was expressed as the 340/380 ratio. The signals were captured and analyzed using the NIS-Elements AR software program (Nikon). All chemicals were directly applied to the bath. The MBP_84-104_ or SCR peptide (5 or 10 μg/ml, each) was administered to female and male DRG. Where indicated, capsaicin (500 nM) was co-administered. Calcium imaging (ratio of 340/380) was used to record the DRG responses to the MBP_84-104_ or SCR peptide and capsaicin.

### 2.10 Immunofluorescence

Tissues were post-fixed in 4% PFA, rinsed, cryoprotected in a 15–30% sucrose gradient, embedded into optimal cutting temperature compound (Sakura Finetek) in liquid nitrogen, and cut into 10-μm-thick sections. DRG cultures grown on the 15–18 mm coverslips were washed with PBS, fixed in 4% PFA for 20 min, and permeabilized with 0.1% Triton in PBS. Non-specific binding was blocked for 30 min in 5–10% goat serum in PBS at ambient temperature. Slides were incubated with the primary antibodies in 0.1% Tween-PBS (16–18 h at 4 °C) followed by the species-specific Alexa 594-conjugated secondary antibody (red; 1:200; Molecular Probes, 1 h, ambient temperature). For dual-immunofluorescence (IF), the second primary antibody (16–18 h at 4 °C) was followed by the goat anti-species Alexa 488-conjugated secondary antibody (green, 1:200, Molecular Probes, 1 h, ambient temperature). Slides were rinsed in PBS and mounted using a Slowfade Gold antifade reagent containing DAPI (4’,6-diamidino-2-phenylindole, Molecular Probes, #536938). Signal specificity was confirmed by omission of a primary antibody or use of isotype control. The images were acquired using All-In-One Fluorescence Microscope BZ-X700 (Keyence, Itasca, IL).

### 2.11 Immunoblotting

Whole tissue and cell lysate aliquots (3–25 μg total protein) or membrane fractions (27 μl per 1 ml) were analyzed by immunoblotting. The lysates were prepared in Tris-buffered saline (TBS) supplemented with 1% Triton X-100, 10% glycerol, 0.1% SDS, 5 mM EDTA, and the protease and phosphatase inhibitor cocktails. Insoluble material was removed by centrifugation (14,000×*g* for 15 min). In addition, nerves were fractionated by a sucrose density gradient, with the membrane fractions 4–6 and 10–12 defined as buoyant lipid rafts-enriched and non-buoyant heavy membrane fractions, respectively. The samples were separated by 10% Tris-glycine SDS-PAGE (Bio-Rad) and transferred to polyvinylidene difluoride membranes (EMD Millipore). Membranes were blocked in TBS containing 5% non-fat milk (Bio-Rad), incubated with the LXRα antibody in 5% BSA (1:1000, 16–18 h at 4 °C), and then the goat anti-rabbit horseradish peroxidase-conjugated secondary antibody (Cell Signaling Technology, 1 h, ambient temperature). TBS containing 0.05% Tween was used for washes. Blots were developed using SuperSignal enhanced chemiluminescence (Thermo Scientific). Blots were re-probed using anti-GAPDH or flotillin-1 antibodies (see *Reagents and Antibodies*, above). The bands were digitized and quantitated using UVP Biospectrum 810 Imaging System.

### 2.12 *Taqman* qPCR

Total RNA was extracted using Trizol (Invitrogen) and purified on a RNeasy mini column (Qiagen). RNA purity was estimated by measuring the OD260/280 ratio. The samples were treated with RNase-free DNAse I (Qiagen). cDNA was synthesized using a Transcriptor first-strand cDNA Synthesis kit (Roche). Real-time RT-PCR was conducted using Mx3005P™ qPCR System (Agilent) in 25 μl reactions containing *Taqman* Universal PCR Master Mix (Applied Biosystems), cDNA (50 ng), and specific forward and reverse primers (900 nM each) and *Taqman* probes (200–300 nM) for each gene (see Table 1), using GAPDH as the normalizer gene, with a one-step program: 95 °C, 10 min; 95 °C, 30 s; 60 °C, 1 min for 50 cycles (Ko et al., 2016). Relative mRNA and fold-change calculations were performed with the Mx3005P™ software, using the 2(-ΔΔC(T)) method (Livak and Schmittgen, 2001; Pfaffl, 2001).

**Table 1:** Primers: 900 nM; Probes: 200–300 nM.

	Gene	Accession #	
LXRα	*Nr1h3*	NM_031627.2	F: AGGGCTCCAGGAAGAGATGT
			R: CAACTCCGTTGCAGAGTCAG
			Probe: Roche#5 (04685024001)
LXRβ	*Nr1h2*	NM_031626.1	F: TTAAGGAGGAGGTACAGGAGACTG
			R: TTGCGCTCAGGTTCATCC
			Probe: Roche#10 (04685091001)
ERα/ESR1	*Esr1*	NM_012689.1	AB Rn01640372_m1
ERβ/ESR2	*Esr2*	NM_012754.1	AB Rn00562610_m1
ABCA1	*Abca 1*	NM_178095.3	AB Rn00710172_m1
ABCG1	*Abcg 1*	NM_053502.2	AB Rn00585262_m1
GAPDH	*Gapdh*	NM_017008	AB Rn 01775763_g1
α2∂1	*Cacna2d1*	NM_012919	F: CATACTCCAGATTGGCTGGTG
			R: AGTAGCTGCTGGAGAATAGACCA
			Probe: Roche#74(04688970001)
IL-6	*Il6*	NM_012589	F: CCCTTCAGGAACAGCTATGAA
			R: ACAACATCAGTCCCAAGAAGG
			Probe: Roche#20 (04686934001)

### 2.13 Lipidomics analysis

Nerve tissues were homogenized into 500 ml of 10% methanol. Normalization was done by weighing the tissue and protein concentration determined using the Coomassie Protein Assay kit. An internal standard mix of 25-hydroxycholesterol (OHC)-d6, desmosterol-d6, and campesterol-d6 (Avanti Polar Lipids) was added to 200 ml of homogenate. Samples were saponified for 1.5 h at 37 °C with a final concentration of 0.2 N KOH, and then extracted with 500 ml of butanol/methanol (3:1, v/v), heptane/ethyl acetate (3:1, v/v), and 1% acetic acid in water (BUME). Extracts were brought to dryness, taken up in 90% methanol and run on a Waters Acquity UPLC interfaced with an AB Sciex 6500 QTrap mass spectrometer equipped with an APCI probe, using the source settings: Curtain Gas 20, Collision Gas Medium, Ion Spray Voltage 5500, Temperature 400, GS1 25, NC 1. A Phenomenex Kinetex C18 1.7 μM (2.1 mm × 150 mm) column was used for the sample chromatography. A 30 min step gradient was employed using 70/30 acetonitrile/water with 5 mM ammonium acetate as Buffer A and 50/50 acetonitrile/water with 5 mM ammonium acetate as Buffer B with a flow of 0.5 ml/min. The gradient started at 0%B for 2 min, ramped to 10%B over 4 min, 15%B over 9 min, 50%B over 11 min, 100%B over 2 min, then held at 100%B for 2 min. Sterol species were identified by mass spectrometry using 30 MRM (Multiple Reaction Monitoring) in positive mode. Standard curves were obtained in parallel using the identical conditions (Lofgren et al., 2016; Quehenberger et al., 2010). Data analysis was performed with Analyst and Multiquant software packages.

### 2.14 LC-MS/mass spectrometry of MBP_84-104_ pull-down

Unless indicated otherwise, all procedures were performed at 4 °C. Nerve tissues were washed with PBS and then solubilized for 1 h using 50 mM Octyl-β-d-glucopyranoside (Octyl) in the pull-down buffer (50 mM HEPES, pH 8.0, 150 mM NaCl, 1 mM CaCl_2_, 1 mM MgCl_2_). Insoluble material was removed by centrifugation (14,000×*g*; 20 min). Normalization was done by weighing the tissue and protein concentration determined using the Coomassie Protein Assay kit. Lysate aliquots (500 μg total protein; 2 ml, each) were 2-fold diluted using the detergent-free pull-down buffer to reach a 25 mM final concentration of the detergent. To remove the non-specific binders, the samples were precleared for 4 h using the biotin-labeled SCR immobilized on Streptavidin-beads (200 μl, 50% slurry). The fall-through fraction was co-incubated for 16–18 h with the biotin-labeled MBP_84-104_ immobilized on Streptavidin-beads. After extensive washing with 25 mM Octyl in pull-down buffer, the bound material was eluted from the individual WT- and SCR peptide immobilized-beads using 2x reducing SDS-loading buffer. The eluted samples were separated in a 4–12% NuPAGE MOPS gel followed by silver staining. Four individual gel sections discriminating the WT sample from the SCR sample were subjected to in-gel reduction (50 mM DTT, 60 °C, 60 min), alkylation (50 mM iodoacetamide, 40 °C, 45 min in the dark), and digestion using Sequencing Grade Modified Trypsin (Promega; 25 μg/ml, 37 °C). Peptides were analyzed by LC-MS/MS using a Proxeon EASY nanoLC system (Thermo Scientific) coupled to an Orbitrap Elite mass spectrometer (Thermo Scientific). Peptides were separated using an analytical C_18_ Acclaim PepMap column 0.075 × 500 mm, 2 μm particles (Thermo Scientific). The mass spectrometer was operated in positive data-dependent acquisition mode. MS1 spectra were measured with a resolution of 60,000, an AGC target of 1 × 10^6^ and a mass range from 350 to 1400 m/z. Up to 10 MS2 spectra per duty cycle were triggered, fragmented by collision-induced dissociation, and acquired in the ion trap with an AGC target of 1 × 10^4^, an isolation window of 2.0 m/z and a normalized collision energy of 35. Mass spectra were analyzed using MaxQuant software version 1.5.5.1. MS/MS spectra were searched against the *Rattus norvegicus* Uniprot protein sequence and the GPM cRAP sequence database of commonly known protein contaminants. Carbamidomethylation of cysteines was searched as a fixed modification, while oxidation of methionines and acetylation of protein N-terminal were searched as variable modifications. Enzyme was set to trypsin in a specific mode and a maximum of two missed cleavages was allowed for searching. The target-decoy-based false discovery rate filter for spectrum and protein identification was set to 1%.

### 2.15 Transcriptome datasets

Our published RNA-seq dataset (Gene Expression Omnibus (GEO) ID GSE107159) obtained from ipsilateral sciatic nerve (injection site), L4-5 DRG and spinal cord (dorsal quarter) tissues at day 7 after IN MBP_84-104_ or SCR peptide (30 μg in 3 μl in PBS, each) or PBS (3 μl) in C57BL6 mice n = 12/group (6 male, 6 female) (Chernov et al., 2020). Normalized absolute gene expression values (Log2TPMs (transcripts per million reads)) for RNA-seq dataset were used in this study. RNA-seq mapping, normalization, and gene identification score (p-value) statistical analyses were conducted using Illumina analysis workflow with standard parameters in Basespace (Illumina), described previously (Chernov et al., 2020). Heatmap diagrams were prepared using the color scale tool in Excel (Microsoft). Gene subsets were selected based on Gene Ontology annotations (http://geneontology.org).

### 2.16 Statistical analyses

Statistical analysis was performed using GraphPad Prism 8.0 (GraphPad Software, San Diego, CA, USA) by one-way or two-way analyses of variance (ANOVA) for repeated measures for comparing three or more groups, followed by the post hoc Sidak's, Bonferroni, Tukey–Kramer's or Mann Whitney rank sum tests, as indicated in Figure Legends. For parametric statistics, the data was confirmed to be normally distributed first. A two-tailed, unpaired Student's t-test was used for comparing two groups. p ≤ 0.05 was considered significant.

## 3 Results

### 3.1 Bioactive MBP_84-104_ fragment acts via the LXR/RXR

As a result of our prior extensive biochemical, cell biological and functional analyses (Chernov et al., 2018; Hong et al., 2017; Ko et al., 2016; Kobayashi et al., 2008; Lee et al., 2022; Liu et al., 2012; Remacle et al., 2018), we identified the bioactive 84–104 region of MBP proteolytically released between days 3 and 27 post-CCI (Fig. 1A). The MBP_84-104_ region represents an evolutionarily conserved sequence, corresponding to 100% identity between human, rat, mouse, and guinea pig genomes (Fig. 1B). Equal dose IN MBP_84-104_ in mice of both sexes induced female-specific allodynia associated with increased *Cholesterol Biosynthesis* and decreased *Cholesterol Efflux LXR/RXR* signaling in female relative to male nerves (Chernov et al., 2020), which advanced to the segmental DRG and/or spinal cord in females (Liu et al., 2012).

To conduct in-depth assessment of this finding, we hypothesized that upon IN injection in sciatic nerve, MBP_84-104_ peptide interacts and affects the function of one or more components of the LXR/RXR complex (Fig. 1C) at one or more sites ipsilateral to the damaged and/or IN injected neuraxis. The axis represents a complex of an LXR with its obligate partner RXRα and a co-activator (CoA). Upon stimulation with an oxysterol ligand (L) such as 25-OHC or a cholesterol precursor (P) such as desmosterol, LXR binds to LXR response element (LXRE) on a promoter of a target gene including *Abc* cholesterol transporter or *IL-6*, leading, respectively, to induction or suppression of their transcription and expression.

The changes in LXR, RXR and target genes controlled by MBP_84-104_ were assessed using targeted transcriptome analyses of our published RNA-seq dataset (GEO GSE107159) (Chernov et al., 2020). The dataset was obtained at day 7 after IN MBP_84-104_ or SCR peptides (30 μg in 3 μl in PBS, each) or PBS (3 μl) into an intact sciatic nerve. Heatmaps of gene expression (absolute values) are shown in the nerve injection site (Fig. 1D) and the ipsilateral DRG and spinal cord (dorsal quarter, Suppl. Fig. S1). IN MBP_84-104_ caused specific (not observed in SCR or PBS groups) downregulation of LXRα (encoded by *Nr1h3*), its obligate partner, RXRα (encoded *Rxra*), and bile acid farnesoid X receptor (FXR, encoded by *Nr1h4)* genes in the nerve of both sexes (Fig. 1D). LXRβ (encoded by *Nr1h2*) and other RXR genes were expressed constitutively and unaffected by any treatment. IN MBP_84-104_ induced Cacna2d1 gene in nerves of both sexes (Fig. 1D). With the exception of FXR*/Nr1h4*, whose expression was restricted to the nerve, all *Nr1h* and *Rxr* genes were constitutively expressed in DRG and spinal cord and unaffected by any treatment (Suppl. Fig. S1). At the injection site, IN MBP_84-104_ regulated transcription of LXR target genes, *Abc*g*4* cholesterol transporter, apolipoproteins *Apoc2*, *Apoh* and *Apoa2*, and cholesterol hydroxylase *CH25H* responsible for 25-OHC synthesis (Suppl. Fig. S1).

**Fig. 2:**
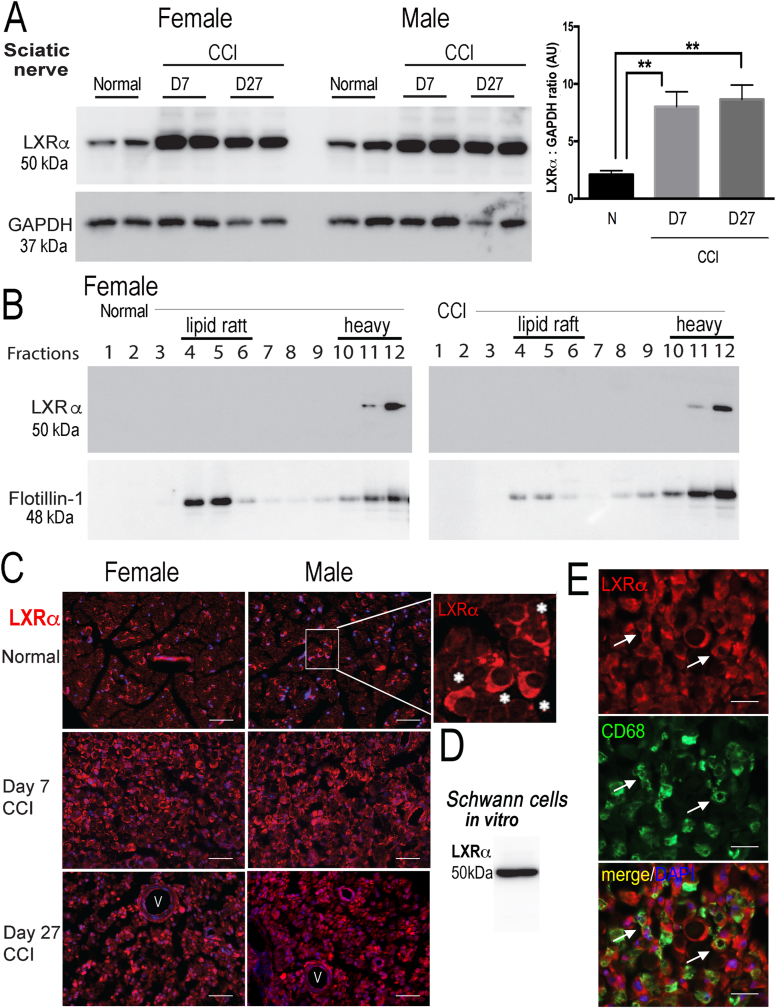
**LXRα in sciatic nerve post-CCI. A-C,** LXRα immunoblotting (50 kDa) in rat sciatic nerves at days 0 (normal), 7 and 27 post-CCI, duplicate sample representative of B (below). GAPDH (37 kDa), a loading control. The graph represents the mean arbitrary units (AU) of LXRα to GAPDH ratio ± SEM of n = 6/group (3 male, 3 female); *, p < 0.05, one-way ANOVA and Bonferroni correction. **B**, LXRα immunoblotting of female nerve sucrose density membrane fractions at days 0 (normal) and 27 post-CCI representing lipid raft buoyant (4–6) and heavy non-buoyant (10–12) fractions. Flotillin-1 (48 kDa), a marker for lipid rafts. **C,** LXRα immunofluorescence (red) in crescent Schwann cells of normal sciatic nerve (inset, asterisks). DAPI (blue). Rep. micrographs of n = 6/group (3 male, 3 female). Scale bars, 50 μm. **D,** LXRα immunoblotting (50 kDa) in primary rat Schwann cell cultures. Rep. of triplicate samples from 3 independent experiments pooled from n = 5/sample). **D**, Dual-IF of LXRα (red) with CD68 (green) in sciatic nerve (female) at day 7 post-CCI display LXRα+ macrophages (yellow, arrows). DAPI stain (blue). Rep. micrographs of n = 3/group. Scale bars, 25 μm. (For interpretation of the references to color in this figure legend, the reader is referred to the Web version of this article.)

**Fig. 3:**
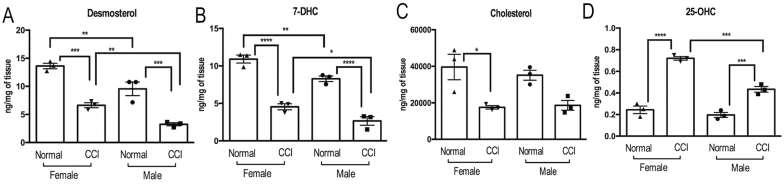
**Sex-specific cholesterol precursor and oxysterol LXRα ligands in sciatic nerve post-CCI**. LXRα ligands using total sterol lipidomics of sciatic nerve at days 0 (sham) and 3 post-CCI. **A**, An intermediate in cholesterol biosynthesis, desmosterol; **B**, 7 carbon atom-modified dehydrocholesterol, 7-DHC; **C,** Cholesterol; **D**, Oxysterol 25-OHC. n = 12/group (6 male, 6 female, pooled 2/sample). One-way ANOVA, multiple comparison test with Sidak's correction (p < 0.05 and 95% CI interval).

**Fig. 4:**
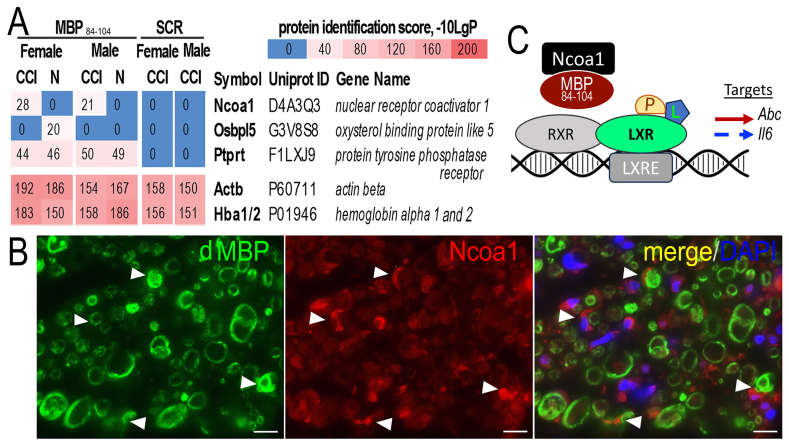
**Ncoa1 and MBP**_**84-104**_**interaction in sciatic nerve post-CCI. A**, LC-MS/MS of MBP_84-104_ or SCR peptide pull-down in rat sciatic nerves at days 0 (sham, N) and 3 post-CCI in n = 6/group (3 male, 3 female). Heat-map of MBP_84-104_-interacting proteins (total of 101 vs SCR), including Ncoa1, Ocbpl5, and Ptprt; Actb and Hba1/2 controls. **B,** Dual-IF for the degraded (d)MBP (AB5864, MilliporeSigma, green) and Ncoa1 (red) in sciatic nerve at day 3 post-CCI (Schwann cells, arrows). DAPI (blue). Rep. micrographs of n = 6/group (3 male, 3 female). Scale bars, 50 μm. **C**, Ncoa1 sequestration by MBP_84-104_ prevents activation of the LXR/RXR axis (a working model). The LXR/RXR axis is detailed in Fig. 1C. (For interpretation of the references to color in this figure legend, the reader is referred to the Web version of this article.)

**Fig. 5:**
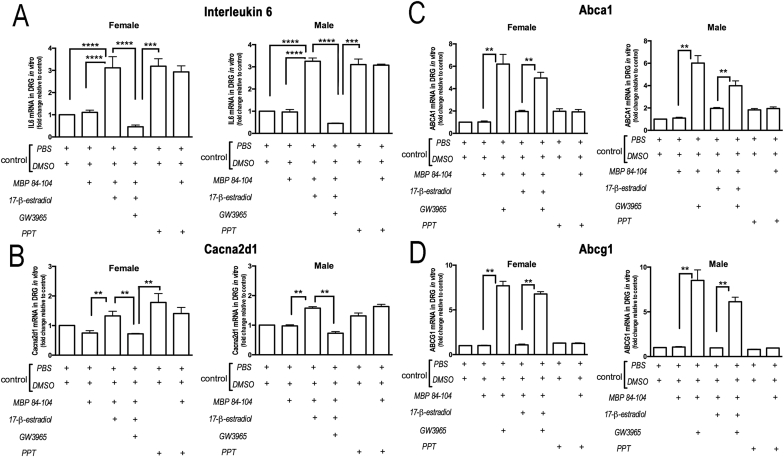
**LXR agonist suppresses effect of estrogens in cultured DRGs.** Taqman qRT-PCR for IL-6 (**A**), Cacna2d1 (**B**), ABCA1 (**C**), and ABCG1 (**D**) in rat DRG cultures treated with 17β-estradiol (100 nM), PPT (100 nM), MBP_84-104_ (10 μg/ml) or PBS for 24 h, followed by LXR agonist (GW3965, 1 μM) or DMSO treatment for 24 h. The mean relative mRNA± SEM of triplicate samples from 3 independent experiments in n = 30 (15 male, 15 female) normalized to GAPDH and compared to PBS or DMSO (**, p < 0.05; ***, p < 0.005; ****, p < 0.0001 by non-parametric Mann Whitney rank sum test). LXR stimulation reversed pronociceptive (Cacna2d1) and proinflammatory (IL-6) effects of 17β-estradiol and PPT in cultured DRGs of both sexes.

**Fig. 6:**
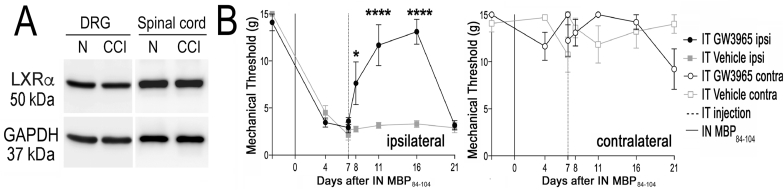
**LXR activation attenuates IN MBP**_**84-104**_**-induced pain. A,** LXRα immunoblotting (50 kDa) in DRG and spinal cord (dorsal quarter) at days 0 (normal) and 1 post-CCI. GAPDH (37 kDa), a loading control. **B**, von Frey testing of ipsilateral (top panel) and contralateral (bottom panel) hind paws after IN MBP_84-104_ (50 μg in 5 μl of PBS), followed by IT injection of LXR agonist (GW3965, 6.2 μg/10 μL, n = 5/group) or vehicle (10 μl, n = 4/group). Mean withdrawal thresholds (g, gram force) ± SEM. Ipsilateral thresholds: two-way ANOVA (****, p < 0.0001, time x treatment; p = 0.0002, time; p = 0.0001, treatment, with Sidak's multiple comparison test (*, p < 0.01)). Contralateral thresholds: two-way ANOVA (p = 0.0079, time x treatment), post hoc tests were without statistical significance.

To further elucidate the role of the LXRα/RXRα axis in female-specific pronociceptive MBP_84-104_ action, we used the model of rat sciatic nerve CCI of both sexes to analyze the changes in LXRα receptor (Fig. 2), its ligands (Fig. 3), and to identify the related MBP_84-104_ interactors (Fig. 4). We then tested the effect of LXR stimulation on MBP_84-104_- and estradiol-induced transcriptional activity in sensory neurons *in vitro* (Fig. 5) and IN MBP_84-104_-induced pain *in vivo* (Fig. 6).

### 3.2 Cholesterol-related LXRα ligand and receptor in sciatic nerve post-CCI

LXRα (50 kDa) immunoblotting was conducted at days 0 (normal), 7 and 27 post-CCI in rat sciatic nerve (injury site, n = 6/group, 3 male and 3 female). GAPDH (36 kDa) was used as a normalizer (Fig. 2A–B). In nerve whole lysates, LXRα was constitutively expressed and significantly elevated at days 7 and 27 post-CCI with no apparent difference between the sexes. To analyze for potential membrane translocation of nuclear LXR (Ishikawa et al., 2013), the nerves were separated into 12 sucrose density gradient membrane fractions: light/buoyant fractions 4–6 defined as lipid rafts (marked by flotillin-1, 48 kDa), and heavy/non-buoyant fractions 10–12. In the normal and CCI (day 7) nerves, LXRα localized exclusively to the heavy non-lipid raft fractions regardless of sex (Fig. 2C, female shown). In sciatic nerve of both sexes, LXRα is produced by Schwann cells *in vivo* (Fig. 2D, normal female nerve shown) and *in vitro* (Fig. 2E). Other LXRα-reactive cells in nerve were endothelial cells and CD68-reactive macrophages (not shown). LXRα levels or distribution showed no statistically significant sex difference in normal or CCI nerve at all time-points.

**Fig. 7:**
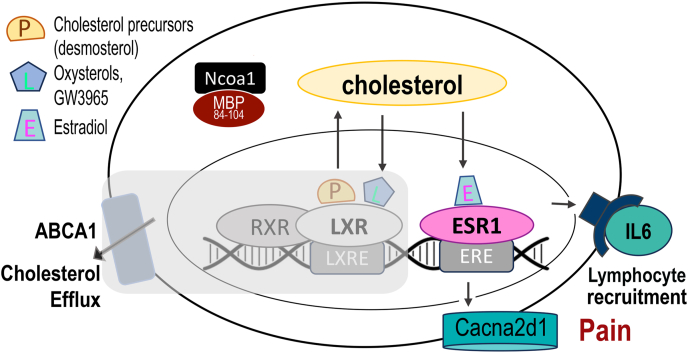
**MBP**_**84-104**_**sustains pain via Ncoa1 sequestration (a hypothesis diagram).** In Schwann cell-axon units of myelinated A-afferent neurons, cholesterol precursor (P) and oxysterol ligands (L) of LXRs control myelin lipid turnover and efflux by activation of the LXR/RXR axis (see Fig. 1C). After nerve injury, MBP_84-104_ released from myelin interacts with Ncoa1, a cytosolic protein co-activator of nuclear receptors. By Ncoa1 sequestration, MBP_84-104_ prevents transcriptional activity of LXR/RXR (grey box) to induce ABCA1/G1 cholesterol transporters and to suppress estrogen/ESR1-induced Cacna2d1 and IL-6 in both sexes. Due to high levels of circulating estrogen, this effect of MBP_84-104_ is prevalent in females. The IN MBP_84-104_ mechanical allodynia is female-specific and mediated by IL-6, Cacna2d1 and lymphocytes (Chernov et al., 2020; Ko et al., 2016; Lee et al., 2022; Liu et al., 2012).

Sciatic nerve lipidomics was used to assess sex- and injury-dependent changes in cholesterol precursor and metabolite LXRα ligands (Fig. 3) in rat sciatic nerves at days 0 (sham) and 3 post-CCI. Two LXRα ligand intermediate precursors in the cholesterol biosynthetic pathway, desmosterol (Fig. 3A) and 7-dehydrocholesterol (DHC, Fig. 3B), reduced post-CCI. Both were elevated in female relative to male nerves at baseline and post-injury. The cholesterol levels followed the patterns of the precursors, but the finding was not statistically significant (Fig. 3C). The levels of 25-OHC, the key oxysterol LXRα ligand, increased post-CCI in nerves of both sexes, with levels significantly elevated in females relative to males (Fig. 3D). Other sterol profiles displayed injury-dependent changes of interest for future in-depth study (Suppl. Fig. S2).

### 3.3 Ncoa1 as an interactor of MBP_84-104_ in sciatic nerve post-CCI

To gain mechanistic insight into the pronociceptive IN MBP_84-104_ action, we set to identify its interactors using LC-MS/MS analysis in the rat sciatic nerve at days 0 (sham) and 3 post-CCI (Fig. 4A). The nerves were lysed, and the non-specific binders were pre-cleared on the biotin-labeled SCR peptide immobilized on Streptavidin-beads. The fall-through material was allowed to bind to biotin-labeled MBP_84-104_ immobilized on Streptavidin-beads. The proteins bound to SCR and MBP_84-104_ columns were eluted, separated by electrophoresis followed by silver staining. The four protein bands distinct in the MBP_84-104_ relative to the SCR sample were excised, digested with trypsin and the tryptic peptides were analyzed by LC-MS/MS.

We identified two protein interactors of MBP_84-104_ in nerve related to the LXR/RXR axis (Fig. 4A). Nuclear receptor coactivator (Ncoa)1 [(or steroid receptor coactivator (Src)1); 2 peptides, K.KDSASASTAMSVSGQAQGSASIK.L and P.VQVTPPR.G; 28% sequence coverage] was observed in the MBP_84-104_ and CCI, but not SCR or normal, nerve samples of both sexes. Oxysterol binding protein like (Osbpl)5 binding was observed in MBP_84-104_, normal female nerve, not SCR, CCI or male nerve samples. Protein tyrosine phosphatase receptor type T (Ptprt) bound to MBP_84-104_, not SCR, in all nerves of both sexes. Actin beta (Actb) and hemoglobin alpha (Hba)1/2 served as non-specific binding controls present in all samples.

Because endogenous MBP_84-104_ release is observed in the injured only nerve (Chernov et al., 2018; Hong et al., 2017; Kobayashi et al., 2008; Lee et al., 2022; Liu et al., 2012; Remacle et al., 2018), we were especially interested in Ncoa1 and its co-localization with the degraded MBP (dMBP) in nerve at day 3 post-CCI. Ncoa1 and dMBP co-distributed within the same myelinated Schwann cell-axon units (Fig. 4B). Clusters of Ncoa1+ cells non-reactive for dMBP were also noted. These data support a model whereby an injury-specific release of MBP_84-104_ may limit the ability of Ncoa1 to activate the LXR/RXR axis (Fig. 4C). This finding alone does not explain the sexual dimorphism of MBP_84-104_ action.

Our findings thus far suggest that sex differences in LXR/RXR action may arise from LXRα ligand, not receptor or co-activator, levels. To test whether LXRα ligand stimulation differentially controls pronociception signaling, we next employed synthetic LXR agonist GW3965 in treatment of cultured DRGs of both sexes.

### 3.4 LXR suppresses pronociceptive estrogen-ESR1 action in cultured DRG of both sexes

LXR stimulation counteracts ESR1 activities in other systems (DuSell et al., 2008). Since 17β-estradiol/ESR1 activation mediates pronociceptive action of certain toxins in DRG neurons (Ferrari et al., 2016), and as also suggested for MBP_84-104_ (Chernov et al., 2020), we next tested the effect of LXR stimulation using synthetic agonist GW3965 on counteracting ESR1 and/or MBP_84-104_ activities in DRG cultures (Fig. 5).

**Suppl. Fig. 3:**
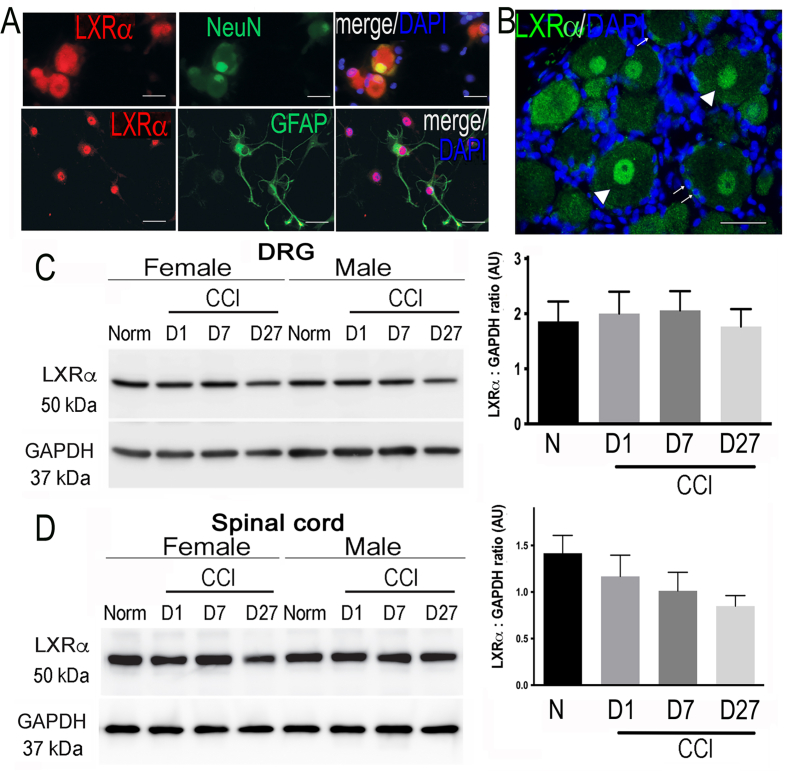
**LXRα in cultured DRG and in DRG and spinal cord post-CCI. A,** Dual-IF for LXRα (red) with NeuN (green) or GFAP (green) or in rat DRG cultures. DAPI (blue). Scale bar, 50 μm. Rep. of triplicate samples from 2 to 3 independent experiments pooled n = 10/sample (5 male, 5 female)**. B,** LXRα IF (green) in rat DRG (CCI, day 7). DAPI (blue). Rep. micrographs of n = 6/group (3 male, 3 female). Scale bar, 50 μm. **C-D**, LXRα immunoblotting (50 kDa) in rat DRG (C) and spinal cord (D, dorsal quarter) at days 0 (normal), 1, 7 and 27 post-CCI. GAPDH (37 kDa), a loading control. The graphs represent the mean arbitrary units (AU) of LXRα to GAPDH ratio ± SEM of n = 6/group (3 male, 3 female; *, p < 0.05; one-way ANOVA and Bonferroni correction.

**Suppl. Fig. 4:**
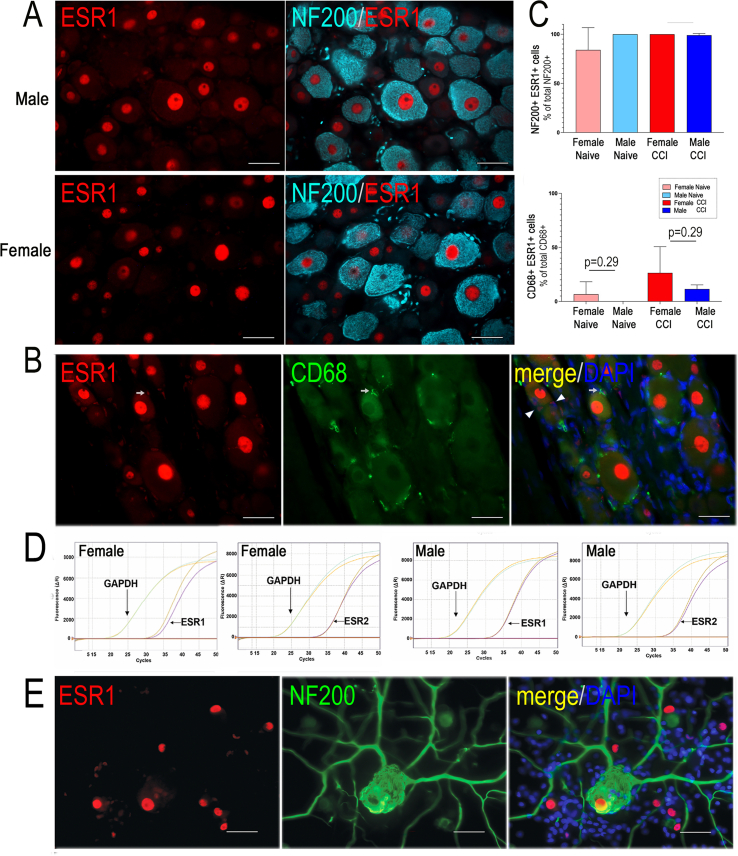
**ESR expression in DRG in vivo and in vitro. A-C,** Dual-IF for ESR1 (red) with NF200 (**A,** green) or CD68 (**B,** green) in rat DRG at day 7 post-CCI. DAPI (blue). Scale bar, 50 μm. Graphs (**C**) represent % ESR1+ of total NF200+ (top) or CD68^+^ (bottom) cells ± SEM from n = 6/group (3 male, 3 female) x 3 areas/n. **D**, Taqman qRT-PCR for ERα/Esr1, ERβ/Esr2 and GAPDH as normalizer in rat DRG cultures. Amplification plots and threshold cycle (Ct) values. **E**, Dual-IF for ESR1 (red) with NF200 (green) in rat DRG cultures. DAPI (blue). Scale bar, 50 μm. D-E: Rep. of triplicate samples from 2 to 3 independent experiments pooled from n = 10/sample (5 male, 5 female).

**Suppl. Fig. 5:**
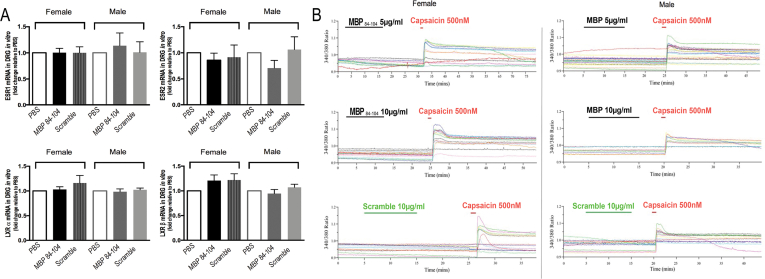
**MBP**_**84-104**_**elicits no effect on LXR and ESR gene expression or calcium signals in cultured DRGs. A,** LXRα/Nr1h3, LXRβ/Nr1h2, Erα/Esr1 and Erβ/Esr2 genes after MBP_84-104_ or SCR peptide (10 μg/ml, each) treatment of rat DRG cultures (grown in DMEM containing 10% FBS for 24 h). The mean relative mRNA± SEM of triplicate samples from 3 independent experiments pooled n = 10/sample (5 male, 5 female) normalized to GAPDH and compared to PBS. **B,** Calcium imaging of dissociated DRG cells loaded with the ratio metric Ca^2+^ indicator dye Fura-2-acetoxymethyl ester. The intracellular calcium concentration (ratio of 340/380) was recorded after MBP_84-104_ or SCR peptide (5 and 10 μg/ml) or capsaicin (500 nM) application for 10 min.

Expression of Erα (*ESR1)*, Erβ (*ESR2*), LXRα (*Nr1h3*), and LXRβ (*Nr1h2*) was confirmed using *Taqman* RT-qPCR, immunofluorescence and/or immunoblotting and showed to be comparable in male and female DRG cultures (Suppl. Figs. 3–5) and not reactive to MBP_84-104_ or SCR treatment regardless of sex (p > 0.05, Suppl. Fig. 5A). ESR1 (Suppl. Fig. 3) and LXRα (Suppl. Fig. 4) nuclear distribution was observed in neurons identified by NeuN (neuron marker) and NF200 (large-diameter neuron marker) *in vivo* and *in vitro*. ESR1 and/or LXRα reactivity in GFAP-positive glia and CD68-positive macrophages was noted. No apparent sex difference in the expression levels or distribution of either nuclear receptor was observed before or after CCI.

The female and male DRG cultures were treated with 17β-estradiol (100 nM, the ESR1/ESR2 agonist) or PPT (100 nM, an ESR1-selective agonist) in the presence of GW3965 (1 μM, LXRα/β agonist) or DMSO vehicle, followed by *Taqman* qRT-PCR for LXR-downstream transporter *Abca1, Abcg1*, and pronociceptive *Il6* and *Cacna2d1* gene expression (Fig. 5), selected as the factors shown to mediate IN MBP_84-104_ induced pain (Chernov et al., 2018; Ko et al., 2016). In a subset of cultures, 17β-estradiol or PPT treatment was done in the presence of the MBP_84-104_ peptide (10 μg/ml). GW3965 treatment was administered 24 h after the estrogens and/or MBP_84-104_. In DRG cultures of both sexes, 17β-estradiol produced a highly significant (p > 0.0001) 3-fold induction of *Il6* (Fig. 5A) and *Cacna2d1* (Fig. 5B) expression. The effect of the PPT to induce *Il6* but not *Cacna2d1* mRNA was statistically significant. GW3965 treatment attenuated the increase in *Il6* and *Cacna2d1* caused by the estrogens. GW3965, but not other, treatments induced *Abca1* (Fig. 5C) and *Abcg1* (Fig. 5D) expression in DRG cultured of both sexes. MBP_84-104_ or SCR peptides produced no significant effect on the expression of either gene.

The effect of MBP_84-104_ treatment on evoked Ca^2+^ transients in rat male and female DRG neurons was assessed using intracellular calcium imaging after MBP_84-104_ (5 and 10 μg/ml) or SCR (10 μg/ml) peptide application for 10 min. Responsiveness to the algogenic compound capsaicin (500 nM) was used for control. The capsaicin sensitivity is evident by the strong calcium signals in the TRPV1-positive DRG neurons of both sexes (Suppl. Fig. 5B). Both MBP_84-104_ and SCR elicited no effect in neurons of both sexes. Due to no observed impact on TRPV1-positive or TRPV1-negative neurons, the changes in the 340/380 ratios were not recordable.

We conclude that DRG neurons of both sexes: (a) express ESRs and LXRs; (b) induce *Il6* and *Cacna2d1* genes upon 17β-estradiol activation via ESR1; (c) block the 17β-estradiol/ESR1 action upon LXR stimulation; (d) induce cholesterol transporter genes upon LXR stimulation; (e) do not change gene expression or intracellular calcium concentration in response to MBP_84-104_.

### 3.5 IT LXR activation attenuates pain induced by in MBP_84-104_

LXR stimulation using GW3965 (6.2 μg in 10 μl of a vehicle) administered IT attenuated mechanical allodynia after sciatic nerve injury by activating spinal and ganglionic LXRα (Xu et al., 2017). Because LXR/RXR related signaling after IN MBP_84-104_ in females progressed from nerve to DRG and/or spinal cord (Chernov et al., 2020; Ko et al., 2016; Liu et al., 2012), we set to test the effect of IT GW3965 in IN MBP_84-104_-induced allodynia.

First, constitutive LXRα (50 kDa) expression (Suppl. Fig. 1) in rat L5 DRG and L1-L6 spinal cord immunoblotting at days 0 (normal) was unchanged at days 1, 7 and 27 post-CCI regardless of sex (Fig. 6A, Suppl. Figs. 3C–D). Mechanical allodynia was established at day 7 post-IN MBP_84-104_ in rat females by the reduced paw withdrawal threshold to von Frey stimulation (Fig. 6B), consistent with our previous reports (Chernov et al., 2018; Ko et al., 2016; Liu et al., 2012). IT GW3965 (6.2 μg in 10 μl of a vehicle) produced a gradual reversal of IN MBP_84-104_-induced allodynia relative to the vehicle (1% DMSO, 4% Tween-80 in PBS, 10 μl) that lasted for over one week. IN injection of SCR peptide or PBS produced no change in paw withdrawal (Chernov et al., 2018, 2020; Hong et al., 2017; Ko et al., 2016; Liu et al., 2012).

## 4 Discussion

Myelin sheath provides insulation necessary to saltatory conduction of Aβ/Aδ afferent neurons (Sherman and Brophy, 2005). In a series of prior works, we have established that demyelination contributes to neuropathic pain in part through T cell-mediated autoreactivity of MBP fragments, such as MBP_84-104_, proteolytically released after a focal nerve trauma (Chernov et al., 2018; Hong et al., 2017; Kobayashi et al., 2008; Liu et al., 2012; Remacle et al., 2018).

We have previously demonstrated that IN MBP_84-104_ induced female-specific allodynia and have attributed it to a 3-tier process of pronociceptive transcriptional remodeling (Chernov et al., 2020). Comparable at the nerve injection site of both sexes, it progressed to the segmental DRG and spinal cord selectively in females. The aim of the present study was to elucidate the role of sex-dependent changes in the nerve that progressed into the DRG and/or spinal cord, including female-specific cholesterol accumulation due to reduced *Cholesterol Efflux*, increased *Cholesterol Biosynthesis* signaling, as well as impaired LXR/RXR signaling and its downstream IL-6 in females (Chernov et al., 2020; Ko et al., 2016; Liu et al., 2012).

Cholesterol homeostasis in peripheral nerve is controlled by the heirarchial transcription factor network that includes LXRs and RXRs (Bookout et al., 2006). IN MBP_84-104_ initiated a manifold control of nerve cholesterol homeostasis. First, IN MBP_84-104_ repressed expression of LXRα and RXRα at the nerve injection site of both sexes. This finding was not recapitulated in the damaged nerves, where LXRα levels were elevated in both sexes. This discrepancy can be potentially explained by high contribution of LXRα-expressing macrophages in the injured nerve, that are not recruited in the nerve post-IN MBP_84-104_ (Liu et al., 2012).

MBP_84-104_ may regulate the synthesis of cholesterol precursor and oxysterol LXRα ligands. Thus, IN MBP_84-104_ induced the expression of 7-dehydrocholesterol reductase (DHCR7), which converts cholesterol precursor 7-DHC to cholesterol, particularly, in female nerves (Chernov et al., 2020). Endogenous MBP_84-104_ release may contribute to prevalence of 7-DHC in injured nerve of females relative to males. Similarly, the injury-induced increase in 25-OHC oxysterol in female correlates to the ability of MBP_84-104_ to induce cholesterol hydroxylase CH25H involved in 25-OHC synthesis (Chernov et al., 2020).

Further, the nuclear receptor co-activator Ncoa1, also known as Src1, emerged as an injury-specific interactor of MBP_84-104_ in nerve of both sexes. By interacting with ligand-bound LXRs and RXRs, Ncoa1 represses expression of inflammatory genes and induces expression of anti-inflammatory genes (Cermenati et al., 2010). Ncoa1 serves to activate a large number of ligand-controlled nuclear receptors, including DNA-bound ESR1 stabilized by estrogens (Glass and Ogawa, 2006) and IN MBP_84-104_-controlled FXR, vitamin D receptor (VDR), peroxisome proliferator-activated receptor (PPAR) (Chernov et al., 2020; Ko et al., 2016; Liu et al., 2012). Thus, through the relationship with Ncoa1, MBP_84-104_ likely controls activities of several nuclear receptors of the hierarchical transcription factor network, including sex steroid receptors.

The role of estrogens in pain is multifold (Amandusson et al., 1995; Khomula et al., 2017; Papka et al., 1997, 2001; Tran et al., 2017, 2020; Vanderhorst et al., 2005). Activity of ESR1 in DRG neurons is thought to augment toxin-induced Ca^2+^-dependent ER-stress (Ferrari et al., 2016). Accordingly, IN MBP_84-104_ activated both ER-stress and ESR1 signaling exclusively in female DRG and spinal cord, and their inhibition by IT administered IP3R inhibitor attenuated IN MBP_84-104_-induced allodynia (Chernov et al., 2020). ESR1 expression and its stimulation by estrogens, including ESR1-specific PPT, induced IL-6 and Cacna2d1 expression in DRG neurons of both sexes. In cells expressing both ESRs, such as DRG neurons (Suppl. Fig. 4a) and (Taleghany et al., 1999), ESR2 serves to dampen transcriptional activity of ESR1 (Coulombe et al., 2011; Ji et al., 2011).

No sexual dimorphism was observed in MBP_84-104_ release (Lee et al., 2022), interaction with Ncoa1 or regulation of LXR post-CCI. Sex- and injury-dependent changes in LXR ligands likely determine sex specificity and degree of anti-nociceptive LXR/RXR action. In support of this assumption, treatment using a synthetic LXR ligand agonist GW3965 attenuated IN MBP_84-104_ -induced mechanical allodynia in females at the dose and route of delivery shown efficacious in a male model of peripheral nerve trauma (Xu et al., 2017). IT delivery of GW3965 is expected to activate both LXRs constitutively expressed in DRG and spinal cord. Yet activity of LXRα, not LXRβ, is believed to prevent the development of mechanical allodynia. Anti-nociceptive activity of LXR stimulation limits ER-stress and fosters cholesterol efflux in DRG neurons (Bao et al., 2017; Cermenati et al., 2010; Gavini et al., 2018; Li et al., 2019; Xu et al., 2017).

Based on the present findings, we propose a model whereby LXR/RXR induces Abc cholesterol transporter and represses estrogen/ESR1-induced Cacna2d1 and IL-6 gene transcription in myelinated Schwann cell-A-afferent neuron units of both sexes. By Ncoa1 sequestration, MBP_84-104_ blocks transcriptional LXR/RXR activity thus fostering pronociceptive effects of ESR1. Due to high levels of circulating estrogens in females relative to males, this effect of MBP_84-104_ is prevalent in females (Fig. 7).

Conversely, low circulating estrogens in males result in low activity of ESR1 in DRG neurons. The injury- and sex-related differences in cholesterol precursors and metabolites suggest the importance of future studies of sterol lipid metabolism in the somatosensory neuraxis, including local synthesis of estrogens in DRG and spinal cord (Tran et al., 2017) and role of oxysterols in binding and partial inactivation of ESR1 (DuSell et al., 2008). Importantly, at comparable treatment doses and conditions, LXR stimulation counteracted pronociceptive activity of estrogen/ESR1 in cultured DRGs of both sexes, consistent with the model that LXR and ESR ligand levels determine sex specificity and degree of anti-nociceptive LXR/RXR action.

Ultimately, MBP_84-104_ is an immunodominant epitope implicated in autoimmune demyelinating disease (Harauz and Boggs, 2013). IN MBP_84-104_ allodynia is mediated by ganglionic and/or spinal IL-6 (Ko et al., 2016) and depends on adaptive immune activity. Thus, T cell activation is comparable in nerves of both sexes exposed to IN MBP_84-104_; while localized to nerves in males, T cells are recruited into DRG and spinal cord exclusively females (Chernov et al., 2020). T cell-deficient athymic nude female rats fail to develop allodynia after IN MBP_84-104_ (Liu et al., 2012).

The endogenous MBP_84-104_ epitope release in CCI nerves of both sexes leads to female-specific circulation of anti-MBP_84-104_ IgM autoantibodies, indicating sex-dependent B cell action (Lee et al., 2022). Although degenerative changes in nerves post-IN MBP_84-104_ are absent and expression of major proinflammatory cytokines is unchanged relative to the scrambled peptide (Chernov et al., 2018, 2020; Hong et al., 2017; Ko et al., 2016; Liu et al., 2012), the role of innate immune activity in IN MBP_84-104_ -induced allodynia cannot be ruled out. This immune cell- and myelin-dependent (Liu et al., 2012; Shubayev et al., 2016) pronociceptive action of IN MBP_84-104_ *in vivo* was not recapitulated in the dissociated unmyelinated DRG cultures.

Because the MBP_84-104_ sequence is identical in human, mouse and rat, we expect our findings in rodent models to be of relevance to traumatic, autoimmune and other neuropathies in humans that feature a targeted MBP_84-104_ release. Thus, using the same assay we identified anti-MBP_84-104_ autoantibodies in female rats post-CCI and in women with multiple sclerosis pain, fibromyalgia (Remacle et al., 2018) and low back radiculopathy (Schuster et al., 2022). Due to its structural homology with acetylcholine M2 receptor and a human coronavirus CoV-OC43 p65-like protein, we argue that MBP_84-104_ contributes to complex regional pain syndrome, viral and idiopathic neuropathies (Ko et al., 2016; Liu et al., 2012; Shubayev et al., 2016, 2018, 2022).

In conclusion, bioactive MBP fragments released in the damaged PNS regulate cholesterol and sex steroid metabolisms by interaction with a nuclear receptor coactivator, Ncoa1. Restoring LXR/RXR activity presents a therapeutic strategy in management of neuropathic pain induced by bioactive MBP. Elevated levels of cholesterol precursors in female relative to male suggest a future study of fundamental sexual dimorphism in sterol composition of peripheral nerves.

## CRediT authorship contribution statement

**Swathi K. Hullugundi:** Conceptualization, Data curation, Formal analysis, Methodology, Validation, Writing – original draft, Writing – review & editing. **Jennifer Dolkas:** Formal analysis, Investigation, Methodology, Validation, Writing – review & editing. **Andrei V. Chernov:** Data curation, Formal analysis, Investigation, Methodology, Writing – review & editing. **Tony L. Yaksh:** Data curation, Funding acquisition, Methodology, Project administration, Supervision, Writing – review & editing. **Kelly A. Eddinger:** Data curation, Formal analysis, Investigation, Writing – review & editing. **Mila Angert:** Formal analysis, Methodology, Writing – review & editing. **Glaucilene Ferreira Catroli:** Data curation, Formal analysis, Investigation, Writing – review & editing. **Alex Y. Strongin:** Data curation, Funding acquisition, Methodology, Project administration, Writing – review & editing. **Patrick M. Dougherty:** Data curation, Investigation, Methodology, Writing – review & editing. **Yan Li:** Data curation, Formal analysis, Investigation, Methodology, Writing – review & editing. **Oswal Quehenberger:** Data curation, Investigation, Methodology, Supervision, Writing – review & editing. **Aaron Armando:** Data curation, Formal analysis, Investigation, Methodology, Writing – review & editing. **Veronica I. Shubayev:** Conceptualization, Data curation, Formal analysis, Funding acquisition, Investigation, Methodology, Project administration, Resources, Supervision, Writing – original draft, Writing – review & editing.

## Declaration of competing interest

The authors declare that they have no known competing financial interests or personal relationships that could have appeared to influence the work reported in this paper.

## Figures and Tables

**figs1:**
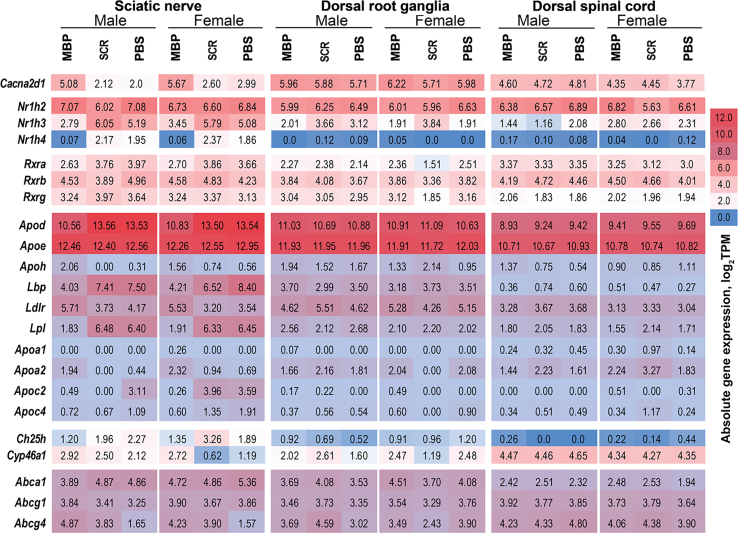


## Abbreviations:

Abca1: ATP binding cassette subfamily A member 1 transporter
Abcg1: ATP binding cassette subfamily G member 1 transporter
Cacna2d1: voltage-gated calcium channel subunit alpha-2 delta-1
CCI: chronic constriction injury
DRG: dorsal root ganglia
ER-stress: endoplasmic reticulum stress
ESR: estrogen receptor
GAPDH: glyceraldehyde 3-phosphate dehydrogenase
2-OHC: 2-hydroxycholesterol
IL-6: interleukin 6
IN: intraneural
IT: intrathecal
LXR: liver X receptor
MBP: myelin basic protein
Ncoa1: nuclear receptor coactivator 1
PBS: phosphate buffered saline
PNS: peripheral nervous system
PPT: 4,4′,4'-(4-Propyl-[1H]-pyrazole-1,3,5-triyl) trisphenol
RXR: retinoid X receptor
Src1: steroid receptor coactivator
